# Dementia and COVID-19 pandemia: The situation in various European countries

**DOI:** 10.1192/j.eurpsy.2021.112

**Published:** 2021-08-13

**Authors:** G. Stoppe

**Affiliations:** Practice-counsel-research, MentAge, Basel, Switzerland

**Keywords:** dementia, COVID-pandemia, nursing homes, Survey

## Abstract

COVID-19 pandemia means a special threat to elderly patients in nursing homes. Dementia sufferers, who make up most of nursing home clients throughout Europe, have been in a critical situation. They bear a higher risk of delirium when affected by the virus. They often do not understand and easily forget, how to use a mask and keep the distance required. In many institutions the elderly were isolated and could not even take their meals together. And finally they do not recognize and even fear nursing staff and other personnel, which has to wear “protective clothes”. Caregivers were told not to visit their loved ones any more.

Where available, modern techniques werde used. Skype and/or Zoom, Facetime telephone should replace face to face contacts. Some insitutions offered visitor rooms, where clients and visitors were separated by acrylic windows and microphones were applied. In some areas, physicians’ visits were reduced to a minimum.

Just recently, regular testing of staff and clients in nursing homes has been introduced. However, this is consuming staff time, which - again - is taken from the patients. We discuss, whether the elderly and their caregivers could set their own preferences.

**Disclosure:**

No significant relationships.

